# Multi-laboratory validation of a Δ9-tetrahydrocannabinol LC-MS/MS test kit designed for quantifying THC and marijuana metabolites in blood

**DOI:** 10.15761/FSC.1000125

**Published:** 2018-03-28

**Authors:** Amy L Patton, Joseph O Jones, Anne Nord, DW Eversole, Erin E Feazell, Kristen Mauldin, Lingyun Li, Lee D Williams, Shasha Bai, Kermit Channell, Gregory Endres, Matthew Gamette, Jeffery H Moran

**Affiliations:** 1 PinPoint Testing, LLC, Little Rock, Arkansas, 72202, USA; 2 Ohio State Highway Patrol Crime Laboratory, Columbus, Ohio, 43209, USA; 3 Idaho State Police Forensic Services, Meridian, Idaho, 83642, USA; 4 Kentucky State Police Central Forensics Laboratory, Frankfort, Kentucky, 40601, USA; 5 West Virginia State Police Forensic Laboratory, South Charleston, West Virginia, 25309, USA; 6 Arkansas State Crime Laboratory, Little Rock, Arkansas,72205, USA; 7 Wadsworth Center, Department of Health, Albany, New York, 12201, USA; 8 Biotage GB Limited, Distribution way, Cardiff, CF82 7TS, UK; 9 Biostatistics Program, Department of Pediatrics, University of Arkansas for Medical Sciences, Little Rock, Arkansas, 72205, USA; 10 Department of Pharmacology & Toxicology, College of Medicine, University of Arkansas for Medical Sciences, Little Rock, Arkansas, 72205, USA

## Abstract

Marijuana legalization has increased the demand for testing of Δ9-tetrahydrocannabinol (THC) and THC metabolites. The THC ToxBox^®^ test kit (THC ToxBox^®^) is commercially available and supports high-throughput LC-MS/MS analytical methods designed to quantify low levels of THC and THC metabolites in blood. The purpose of this study is to determine if this new test kit meets the rigors of laboratory accreditation and produces equivalent results across six states- and locally-funded laboratories. Each laboratory followed internal method validation procedures established for their clinical (CLIA) or international (ISO17025) accreditation program. Test performance indicators included accuracy, precision, measurement of uncertainty, calibration models, reportable range, sensitivity, specificity, carryover, interference, ion suppression/enhancement and analyte stability. Analytes and interferents were resolved within the 6-min analytical runtime, and the 48-well plate pre-manufactured with calibrators, second source quality control material, and internal standards at precise concentrations allowed for simple and consistent sample preparation in less than one hour. Every laboratory successfully validated test kit procedures for forensic use. Differences in sensitivity were generally associated with the use of older equipment. Statistical analysis of results spanning reportable ranges show that laboratories with different instrument platforms produce equivalent results at levels sufficiently low enough to support per se limit testing of THC and THC metabolites (1–5 ng/mL). THC ToxBox^®^ represents a viable option for state- and locally-funded laboratories charged with investigating impaired driving cases involving marijuana use.

## Introduction

**Figure 1:**
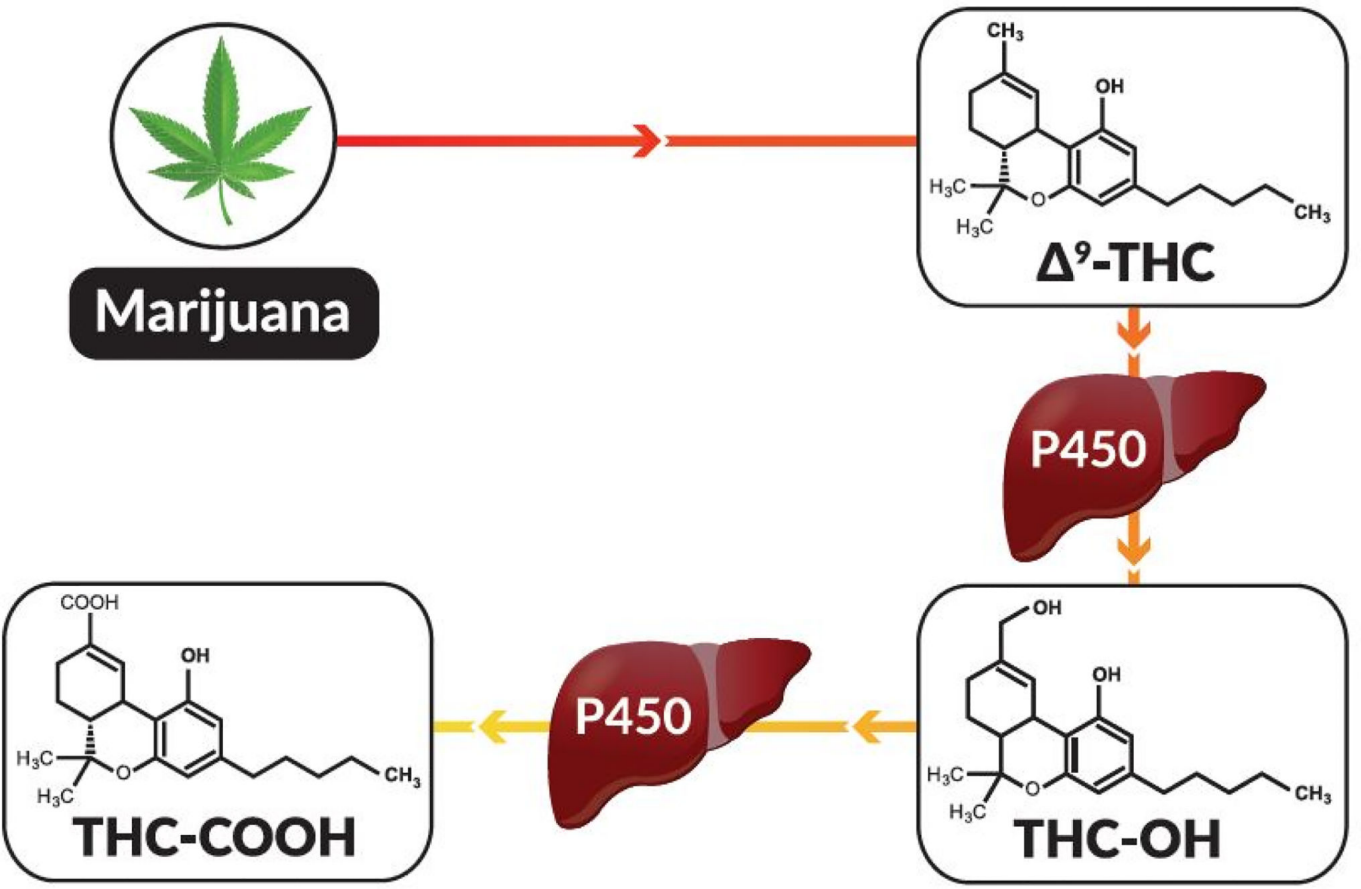
A schematic representation of THC found in marijuana and how THC is metabolized in humans.

Marijuana has been studied extensively since the discovery of its primary active constituent Δ9-tetrahydrocannabinol (THC) (Figure 1) in 1964. It is well documented that peak plasma concentrations of Δ9-THC occur rapidly in human studies using oral and inhalation routes of administration [1,2]. Rapid distribution and absorption of Δ9-THC into fat quickly reduces blood concentrations [3,4], and in the liver, Δ9-THC is quickly metabolized by cytochrome oxidative pathways that give rise to the THC-OH and THC-COOH metabolites [3] (Figure 1). Thirty to sixty minutes after smoking or ingesting marijuana, THC-COOH concentrations are greater than Δ9-THC concentrations [1,4,5]. This pharmacokinetic feature of Δ9-THC is an important consideration for per se limit testing platforms, because it infers that the ratio of Δ9-THC to THC-COOH is proportional to the exposure window. Clinical studies are lacking to conclusively correlate level of cognitive impairment with blood concentrations of Δ9-THC in humans [6], although some reports indicate humans show signs of impairment when Δ9-THC or THC blood metabolite levels reach 2 to 5 ng/mL [7]. As a result, driving regulations in most states have adopted either a no-tolerance or near no-tolerance per se limit.

Most states establish THC per se limit laws ranging from 1 ng/mL to 5 ng/mL. Colorado, for example, has a ‘Reasonable Inference’ level of 5 ng/mL for THC. In 2013, the National Safety Council’s Alcohol, Drugs and Impairment Division (NSC-ADID) suggested similar laboratory guidelines [8], and the International Drug Evaluation and Classification (IDEC) Program recommended a 1.0 ng/mL confirmation level for THC and THC-OH and a 5.0 ng/mL confirmation level for THC-COOH. These low-level targets in blood routinely challenge detection using some of the best technology available to forensic laboratories.

Recent advances to detect low levels of THC and THC metabolites in biological samples use liquid chromatography (LC) [9–11] and gas chromatography (GC) [12–14] techniques. The precise methodology varies greatly between studies, but in general, low limits of detection (LOD) at or below 5.0 ng/mL have been reported. GC/MS analysis in particular involves long run times, and complicated derivatizations and extraction methods. LC-MS/MS methods do not require derivatization and are reported to streamline testing. Blount et al. recently described an ultra-low-level LC-MS/MS method validated for biomonitoring of Δ9-THC and THC metabolites [11]; however, this method was only validated for urine and requires the use of sensitive high-end mass spectrometry not readily available to most forensic laboratories.

A standardized approach for testing Δ9-THC and THC metabolites in blood is needed by state and locally-funded laboratories challenged with the increasing pressures of marijuana legalization. The method must meet strict accreditation requirements at low target levels (1 to 5 ng/mL). The objective of the present study was to validate a commercially available THC ToxBox^®^ test kit (THC ToxBox^®^), which is specifically designed to support forensic laboratories interested in establishing high throughput analytical methods to quantify low levels of THC and THC metabolites in human blood.

## Experimental section

### Reagents and chemicals

**Figure 2:**
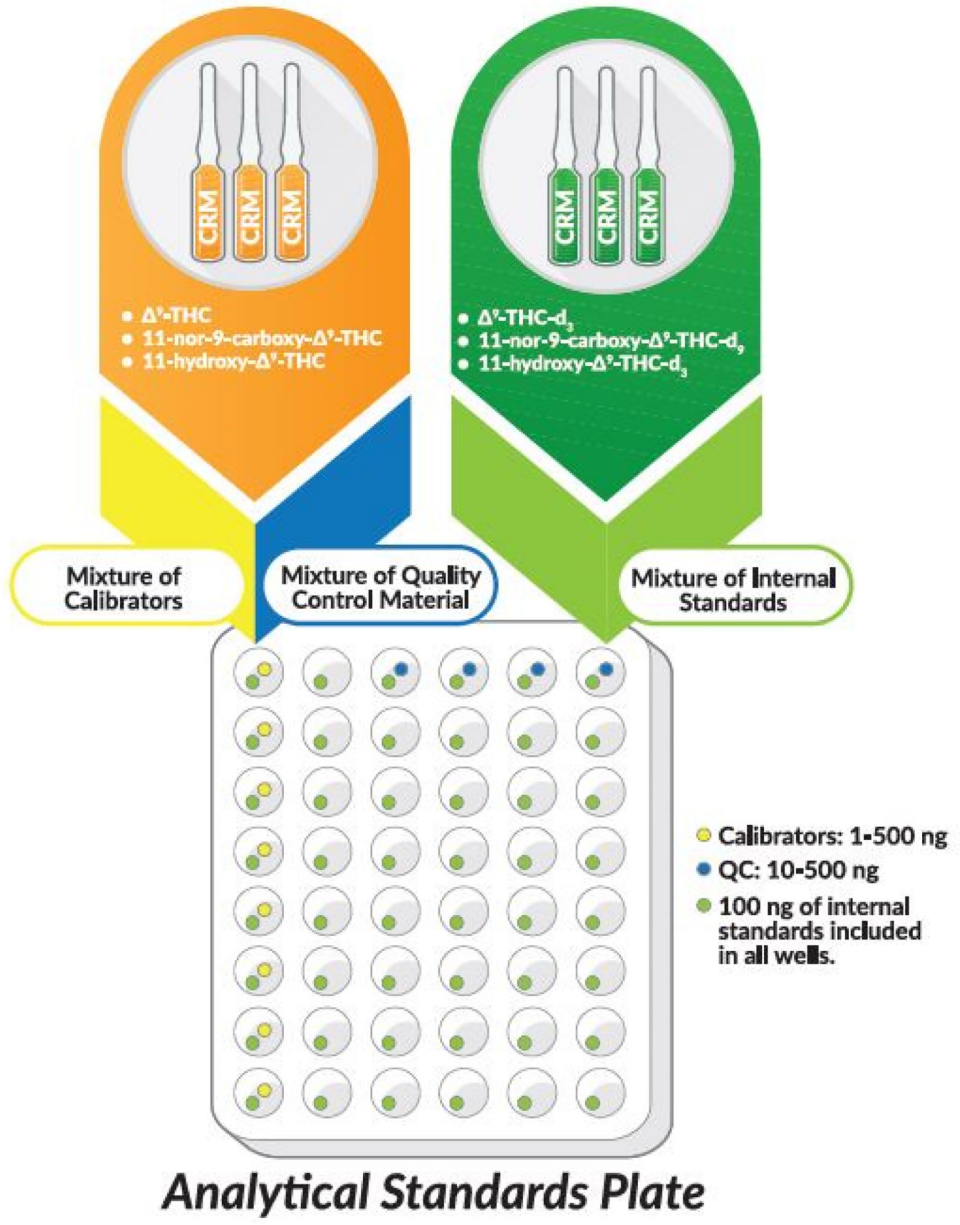
A schematic representation of the commercially available ToxBox^®^ forensic test kit. Each test kit is manufactured with NIST-traceable certified reference material. First and second source material is used for calibrators and quality control samples, respectively. Calibrators range from 1 to 500 ng/mL per well after addition of 1 mL of blank matrix. Second source quality control samples range from 10 to 500 ng/mL per well after addition of 1 mL of blank matrix. All wells contain 100 ng/mL of each isotopically-labeled internal standard (THC-d3, THC-OH-d3, and THC-COOH-d9) after addition of 1 mL of matrix. (Certain elements of this figure were taken with permission from www.caymanchem.com).

**Figure 3:**
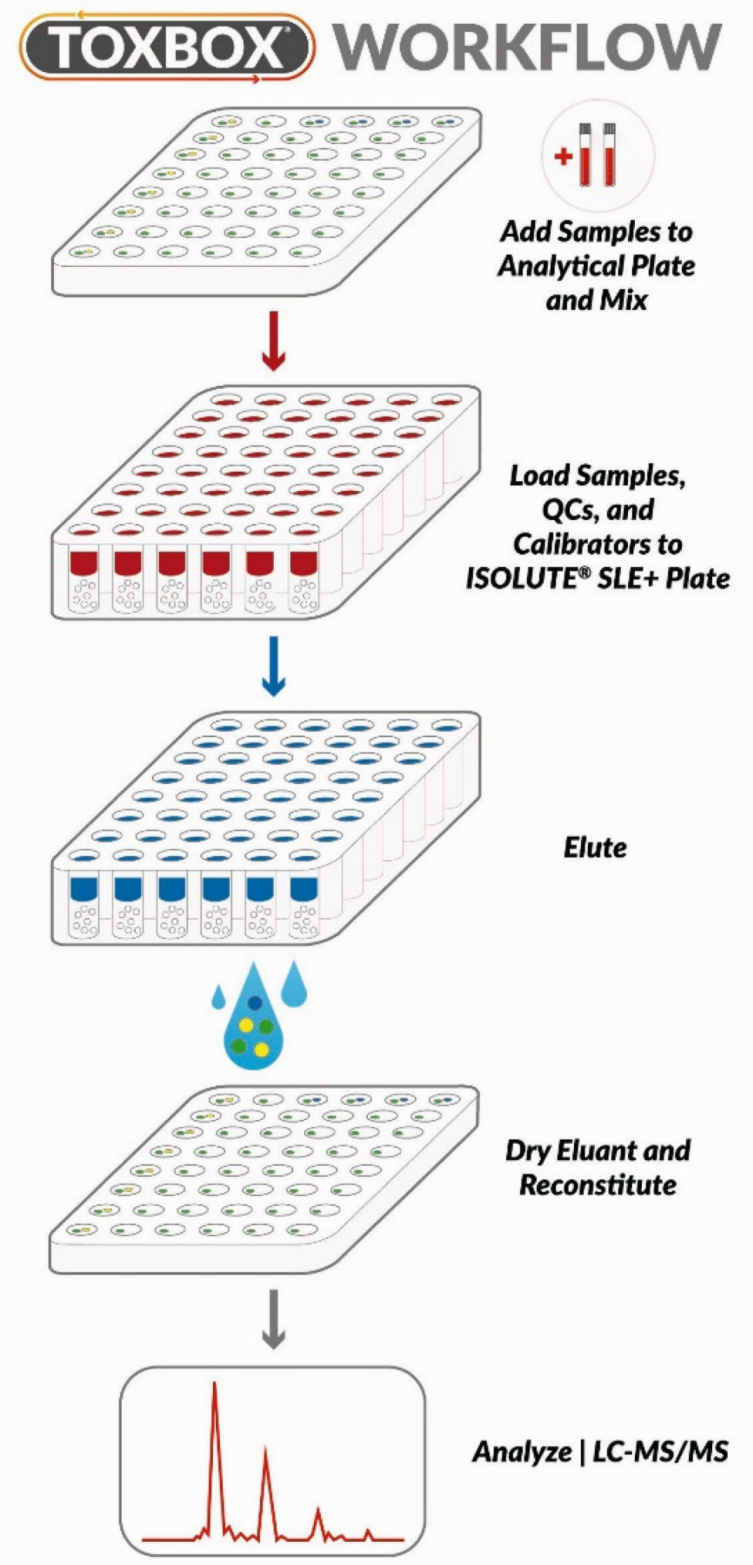
A schematic representation of the ToxBox^®^ forensic test kit workflow. The package insert included in the ToxBox^®^ provides a simplified workflow appropriate for high-throughput testing strategies. Either blank matrix or unknown specimens are pipetted in appropriate wells, mixed, and loaded in ISOLUTE® SLE+ 48-wellplate under gentle vacuum or positive pressure. Samples are then eluted with MTBE (2.25 mL) followed by hexane (2.25 mL). Eluent is evaporated to dryness and reconstituted with 100 µl methanol and analyzed using optimized LC-MS/MS procedures (see *Experimental Section* for details). (Certain elements of this figure were taken with permission from www.caymanchem.com).

The commercially available THC ToxBox^®^ kit provided by Cayman Chemical Company (Ann Arbor, MI) or PinPoint Testing, LLC (Little Rock, AR) streamlines sample preparation and testing procedures to allow high-throughput testing capacity (Figures 2 and 3). This kit incorporates NIST-traceable, certified reference material for all standards and isotopically-labeled internal standards (THC-d3, THC-OH-d3, and THC-COOH-d9) to control for extraction efficiencies. The kit also includes ISOLUTE^®^ SLE+ 48-well plates manufactured by Biotage (Charlotte, NC). Optima-grade formic acid, acetonitrile, and methanol were purchased from Fisher Scientific (Fairlawn, NJ). Deionized water was purified to 18.2 MΩ•cm resistivity using the equivalent of a Millipore laboratory water purification system. All other chemicals and supplies were provided by Cerilliant (Round Rock, TX), Cayman Chemical Company, Biotage or HemoStat Laboratories (Dixon, CA). Blank defibrinated sheep blood or blank human blood void of THC, THC-OH, and THC-COOH contamination was used for all studies.

### Equipment

Initial validation studies used supported liquid extraction (SLE) optimized for 48-wellplate processing on a PerkinElmer Zephyr G3 SPE Workstation (Waltham, MA). Sample extracts were analyzed using an Agilent 1260 quaternary liquid chromatography system (Santa Clara, CA) coupled to an Agilent 6420 tandem mass spectrometer (LC-MS/MS). Instrument control and data acquisition relied on MassHunter LC/MS Data Acquisition (VER B.08.00). Data analysis was performed using MassHunter Quantitative Analysis (VER B.07.01 SP2). Specific equipment used for inter-laboratory comparisons varied between different testing facilities (Supplemental Tables S1 – S5).

### Preparation of analytical standards and quality control material

Analytes of interest for this study included THC, THC-OH and THC-COOH (Figure 1). Analytical standards of each analyte and second source quality control material used for these studies were provided in the THC ToxBox^®^ kit (Figure 2). Standards, second source QCs, and internal standards are manufactured in a 48-wellplate format to deliver precise concentrations, as described in package inserts.

Prior to analysis, drug residue in each well is reconstituted in 1.0 mL of whole blood to build analytical standards (1 ng/mL to 500 ng/mL) and second source QCs spanning the linear working range (10 ng/mL, 25 ng/mL, 100 ng/mL, and 500 ng/mL) (Figure 3). Internal standards also are premanufactured in each standard and QC well in addition to blank wells for unknown specimen analysis. The final internal standard concentration in 1 mL blood samples was 100 ng/mL for THC-d3, THC-OH-d3, and THC-COOH-d9, which targeted the midpoint of linear working ranges for the analytes of interest.

### Solid phase extraction of standards, quality control material, and specimens

All blood calibration standards, QC material, and unknown samples were processed identically by mixing 1 ml of blank blood or unknown specimens in appropriate wells at 900 rpm for 15 min, acidifying with 0.5 mL of 0.1% formic acid, and then mixing for another 15 min at 900 rpm (Figure 3). Samples were then loaded, under gentle vacuum or positive pressure, onto a 1 mL ISOLUTE^®^ SLE+ 48-wellplate. Samples were allowed to equilibrate for 5 min before extracting under gravity with 2.25 mL of MTBE (Methyl tert-butyl ether). A second extraction using 2.25 mL of hexane under gravity followed collection of MTBE extracts. Slight pressure or vacuum was used to remove any residual MTBE or hexane from the sorbent material. All eluent, MTBE and hexane, was collected in deep 48-wellplate reservoirs and evaporated to complete dryness at approximately 35°C under a constant flow of nitrogen. Analytes were reconstituted in 100 µl of 100% methanol. Plates were sealed with aluminum foil prior to analysis. All extracts were immediately assayed or stored at 4°C until analysis.

### Liquid chromatography tandem mass spectrometry

The standard THC ToxBox^®^ LC-MS/MS method specified in the package insert utilized 10 µl injections on a 3 µm UCT Selectra DA (100 × 2.1 mm) LC column heated to 50°C. Analytes were resolved at 0.6 mL/min using mobile phase A (0.1% formic acid in ultrapure 18.2 MΩ•cm water) and mobile phase B (0.1% formic acid in acetonitrile). Isocratic conditions (45% mobile phase A/55% mobile phase B) were used for the first 3.5 min of the analytical run. Mobile phases were then ramped to 80% mobile phase B and held constant for 2.0 min to wash the column between each injection. A 2.5 min post-run equilibration period was used to equilibrate the column back to starting conditions prior to the next injection. The total run time including column equilibration period between injections was 7.5 min. Each laboratory used these exact specifications throughout the study. Specific mass spectrometer and analyte parameters are provided in Supplemental Tables S1–S8. Two transitions were monitored for each analyte. Ion ratios were matched to those of calibration standards to ensure interfering metabolites and other compounds were resolved. To ensure carryover was not present, matrix-matched samples containing no calibration standard material were injected, and blanks were injected following analysis of a known high-concentration sample (i.e., high level standards and QCs) and no carryover was detected.

### Human subject study design

De-identified human samples testing positive for THC were used to demonstrate results obtained from authentic human specimens. Use of this material was approved by the Institutional Review Board of the University of Arkansas for Medical Sciences (Little Rock, AR) (IRB #206577).

### Interlaboratory/Mass spectrometer comparison study

Participating laboratories were accredited to CLIA or ISO17025 standards, and included the Ohio State Highway Patrol Crime Laboratory (Columbus, OH), Kentucky State Police Central Forensics Laboratory (Frankfort, KY), Idaho State Police Forensic Services (Coeur d’Alene, ID), West Virginia State Police Forensic Laboratory (South Charleston, WV), Wadsworth Center (Albany, NY), and the Arkansas State Crime Laboratory (Little Rock, AR). The primary difference between testing facilities was the type of mass spectrometer. Thus, specific operating parameters were optimized for each mass spectrometer (Supplemental Tables S1–S8). The Ohio State Highway Patrol Crime Laboratory (Columbus, OH) and the Wadsworth Center (Albany, NY) extracted samples using the PerkinElmer Zephyr G3 SPE Workstation (Waltham, MA). All other participating laboratories manually extracted samples.

### Statistical methods and laboratory accreditation requirements

While each laboratory maintained independent Quality Assurance/Quality Control programs, method validation requirements were similar and generally followed Society of Forensic Toxicology (SOFT) guidelines established for forensic laboratories, international standards typically used to regulate forensic and FDA laboratories (ISO17025), and CLIA standards established for clinical laboratories. When accuracy, precision, measurement of uncertainty, calibration model, reportable range, sensitivity, specificity, carryover, interference, ion suppression/enhancement, and analyte stability met required performance specifications established by each laboratory, method validations were considered successful. Accuracy and precision were determined using QC samples prepared for independent experiments performed over non-consecutive days. Accuracy was calculated as the absolute percent relative error for each of the expected QC concentrations.

(1) % Relative Error=((Calculated Concentration−Expected Concentration))/(Expected Concentration)×100

Analytical precision was calculated as standard deviation (std. dev.) or as the coefficient of variance (%CV) for replicate measurements at three or four QC concentrations spanning the calibration range. Since lower limit of quantification (LOQ) can be calculated multiple ways, LOQs were normalized between all laboratories and calculated as 3 times the standard deviation of the mean recovery of the low-level standard (either 1 or 5 ng/mL). The LOQ was adjusted to higher levels if the estimated level was lower than the limit of detection (LOD). The LOD was defined as the lowest calibrator level that could be confirmed through ion ratio comparisons. Coefficients of determination (r^2^) were calculated to assess linearity of each individual standard curve. A minimum r^2^ > 0.99 was required for passing validation. A one-sample mean-equivalence test was used to determine if all participating laboratories produced equivalent results for second source quality control samples (10, 25, 100, and 500 ng/mL). These concentrations were chosen to span the linear ranges validated by the laboratories. Different from the one-sample t-test, which is more commonly used and where the null hypothesis assumes no difference, the null hypothesis of the equivalence test is that the difference between the calculated and expected concentration is greater than a pre-specified threshold margin [15]. Equivalence testing was completed at each expected concentration separately with a 20% margin at 5% significance level. Equivalence is inferred if both p-values of two one-sided t-test is less than 0.05. All statistical analyses were performed in Microsoft Excel version 2016 (Redmond, WA) or Stata version 14.2 (StataCorp, College Station, TX).

## Results and discussion

Forensic laboratories often are the target of increased scrutiny in the judicial system, and accordingly require strict validation procedures to meet recommendations issued by organizations including SOFT or the Scientific Working Group for Forensic Toxicology (e.g. SWGTOX), and also to meet standards provided by CLIA and international accrediting organizations (e.g. ANAB and A2LA). Decreased discretionary spending, the lack of adequate personnel, and the intensive time required for executing robust validations make it difficult for state- and locally-funded laboratories to sustain complex toxicological analyses. For example, the 2016 DUI laboratory survey conducted by the Center for FSRE shows that 25% of the participants are not meeting guideline recommendations for THC testing, 17% are not reporting THC results, and 41% report the lack analytical capacity and technology as primary reasons for deficiencies [16]. Thus, sustainable solutions that enable a streamlined and standardized approach for THC per se limit testing will become a critical asset as many states legalize medicinal marijuana and other recreational cannabis programs [17–19].

**Figure 4:**
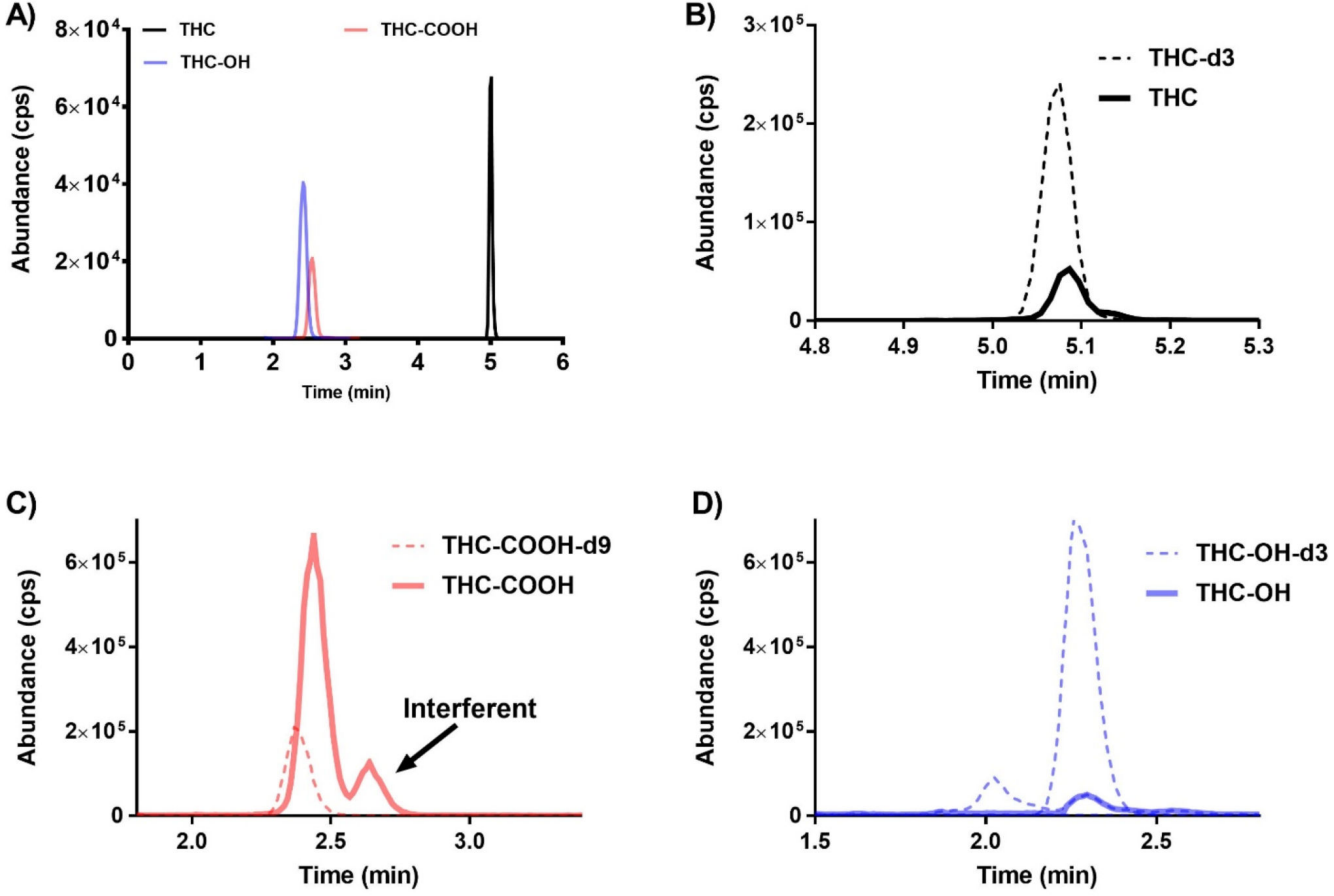
Representative LC-MS/MS chromatographs from (A) a 100 ng/mL quality control sample produced in defibrinated sheep blood and (B – D) a commercially available human sample positive for marijuana use. Chromatography of standards, quality control material, and unknown samples were similar between all sheep and human samples assayed. Different color tracings are representative of the Specific Reaction Monitoring (SRM) experiments used for each specific analyte (see *Experimental Section* for details).

The LC-MS/MS approach presented here achieves baseline separation of THC and the THC metabolites evaluated most commonly in impaired driving cases (Figure 4A – 4D). Chromatography of standards, QC samples, and unknown specimens were similar between all seven participating laboratory sites. Retention times established for each analyte and isotopically-labeled internal standard remained constant (± 0.1 minute) across validation studies. Importantly, the chromatography resolved an interferent that is isobaric to THC-COOH (Figure 4C). This interferent has previously been reported, and it is not known if the interferent is a constituent of the marijuana plant or represents an unidentified metabolic product of THC. It is only found in samples collected from individuals who used marijuana [20]. Blank blood used for method validation does not account for this interferent, and it is critical that laboratories resolve this interferent for accurate measurement of THC-COOH. Several laboratories reported that ion ratios required for THC-COOH confirmation will fail if the interferent co-elutes.

Differing mass spectrometers were the primary difference observed among the testing laboratories. However, key method performance indicators were similar between the laboratories and the different instrument platforms (Tables 1–3). All calibration curves exhibited a high degree of linearity (averaged r^2^ > 0.995). The linear working range for THC, THC-OH and THC-COOH ranged from 1 to 500 ng/mL in most laboratories, but two laboratories using the older mass spectrometers (ABSciex 4000 LC-MS/MS QTrap and Agilent 6410 LC-MS/MS) limited THC, THC-OH, and THC-COOH curves to 250 ng/mL (Tables 1–3). The Wadsworth Center also limited the THC calibration curve because 500 ng/mL fell outside the linear working range of the ABSciex 6600 Triple TOF.

The sample extraction procedure enabled low-level detection on multiple instrument platforms by adequately removing interfering components and background noise (Figure 4A–4D). Depending on the specific analyte and mass spectrometer utilized, limits of detection meet per se limit testing requirements and ranged from 1 to 5 ng/mL. Even though quantifying ions often could be measured at levels lower than the reported LODs, this study required confirmation of the analyte by evaluating secondary confirmation ions at the LOD.

**Figure 5:**
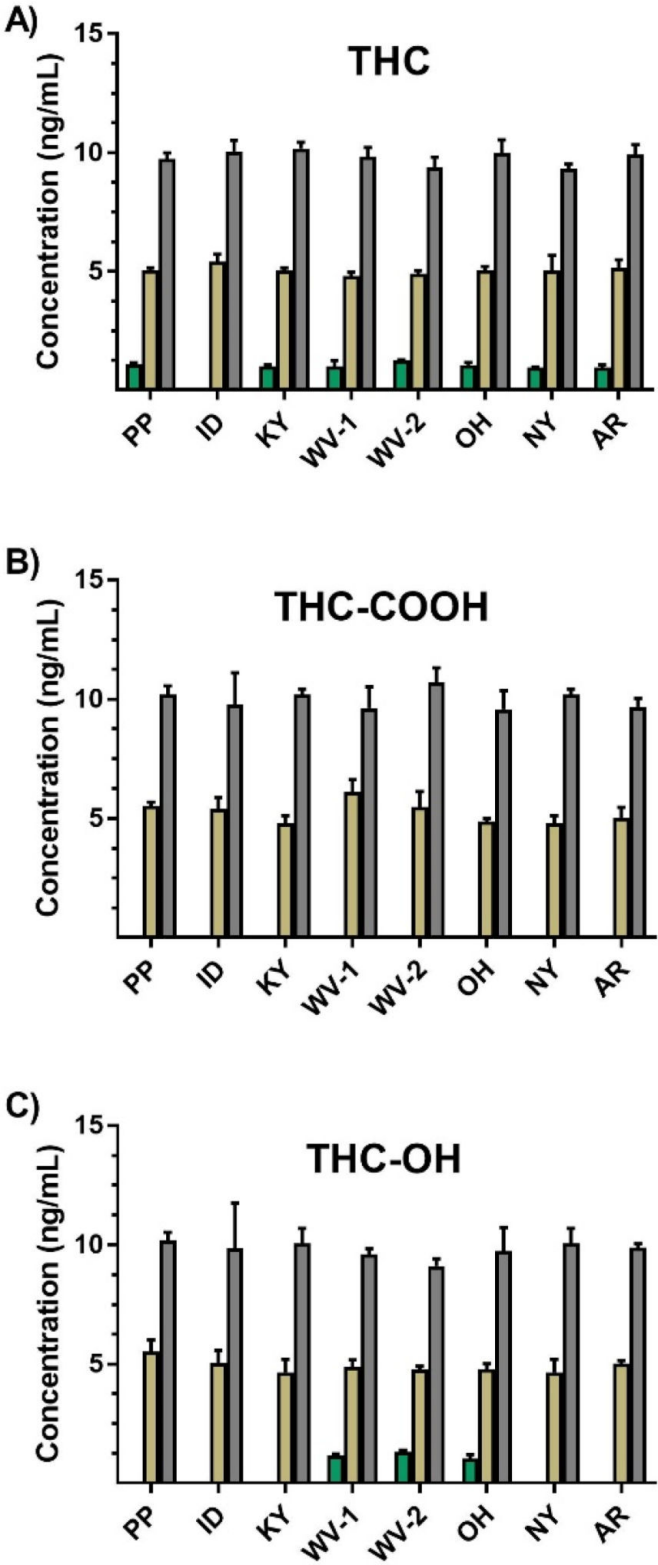
Results from a detection limit study that evaluated the recovery of three low level standards used for (A) THC, (B) THC-COOH, and (C) THC-OH. Green, gold, and silver bars represent 1, 5, and 10 ng/mL standards, respectively. Data are representative of 3 to 6 individual experiments and are presented as mean ± std. dev.

Limits of quantification were assessed by evaluating the recovery of three low level standards (1, 5, and 10 ng/mL) (Figure 5). All laboratories were able to positively identify and adequately measure each metabolite at 5 and 10 ng/mL. The Idaho State Police Forensic Services Laboratory was the only laboratory unable to identify and measure THC at 1 ng/mL. Most likely the loss of sensitivity in this case is directly related to the use of an older Agilent 6410 LC-MS/MS. This system has approximately 10 to 1000 times less sensitivity when compared to the other instrument platforms. No laboratory was able to detect THC-COOH at 1 ng/mL, and only West Virginia State Police Forensic Laboratory and Ohio State Highway Patrol Crime Laboratory were able to detect THC-OH at 1 ng/mL. The West Virginia laboratory performed studies on two different Agilent 6460 LC-MS/MS systems and the Ohio laboratory used an ABSciex 4500 LC-MS/MS QTrap. Interestingly, the Kentucky State Police Central Forensics Laboratory also used the ABSciex 4500 LC-MS/MS QTrap but did not detect THC-OH at 1 ng/mL. The higher-end ABSciex 6600 Triple TOF used by the Wadsworth Center also did not provide adequate sensitivity to reliably detect THC-OH at 1 ng/mL, nor did the Agilent 6420 LC-MS/MS used by the PinPoint Testing, LLC laboratory, or the ABSciex 4000 LC-MS/MS QTrap used by the Arkansas State Crime Laboratory. Estimates of LOQs were normalized among all participating laboratories and ranged approximately from 0.1 to 2.0 ng/mL, which were lower than LODs. Thus, LOQs were administratively defined as the LOD (Tables 1–3).

Not only is it important, but accrediting bodies now often require that forensic laboratories evaluate standards, methods, and practices through second source material studies and interlaboratory comparisons. This method shows that recovery of second source quality control material spanning linear working ranges was similar between laboratories using different testing platforms (Figure 5 and Supplemental Tables S9–S16). A QC high sample along with two QC mid-level samples and a single QC low sample (500, 100, 25, and 10 ng/mL, respectively) were evaluated as long as sample concentrations remained within the laboratory-defined linear working range. Mean percent relative error ranged from 0.7 to 3.5% for THC, −0.2 to 4.0% for THC-COOH, and −0.7 to 2.8% across all concentrations (Table 4). Supplemental Tables S9–S16 provide statistical summaries for each individual laboratory and analyte. Statistical evaluation using a one-sample mean-equivalence test shows that all the participating laboratories produce equivalent results for each measured analyte and concentration (Table 4).

Although the interlab consistency at low levels of THC is strong, the ToxBox^®^ method can always be improved. Currently the THC ToxBox^®^ is restricted to a 48-well plate, and other platforms may need to be explored in instances where increased capacity is needed. Cases requiring ultra-low levels of detection may require the extraction of sample volumes that exceed 48-wellplate capacity. In these instances, larger plates will need to be used or multiple well extracts may need to be combined. In addition, older LC autosamplers may not be able to operate with well plates. These laboratories are forced to place final extracts in autosampler vials, which greatly increases analytical time and costs.

## Conclusions

This is the first study to fully validate the commercially available THC ToxBox^®^ forensic test kit designed to support per se limit testing of Δ9-THC, THC-OH, and THC-COOH. This analytical testing procedure provides for a sustainable, streamlined approach to accurately and reproducibly measure trace amounts of Δ9-THC, THC-OH, and THC-COOH in blood. The unique formulation of the test kits that incorporates pre-manufactured calibrators and quality control material in a ready-to-use format is a first-of-kind for LC-MS/MS. Calibrators, controls, and unknown specimens are made and processed in parallel, which allows for a 48-well plate to be fully processed in about an hour by one analyst. Pre-manufacturing of standards and internal standards not only reduces analytical time, but also provides quality improvement by minimizing scientist-to-scientist and laboratory-to-laboratory variations.

The LC-MS/MS method presented as part of the THC ToxBox^®^ validation is equivalent to previously reported methods, and provides baseline resolution of each analyte, while resolving isobaric interferences in authentic samples. LODs and LOQs also are similar to earlier published methods and are sufficient to meet the analytical requirements for per se limit testing associated with marijuana use. Depending on specific state or internal laboratory requirements, the sensitivity of specific mass spectrometers may need to be considered. Inter-laboratory comparisons establish the validity of this test method in forensic toxicology laboratories and confirms the reliability and robustness of this new technology.

## Supplementary Material

Supplementary Tables

## Figures and Tables

**Table 1:** Summary of linear ranges, correlation coefficients, detection limits, and quantification limits for THC*.

Laboratory/Instrument Comparison for THC					
Instrument	Laboratory	Linear Working Range (ng/mL)	Average r^2^ value	L.O.D.^a^ (ng/mL)	L.O.Q.^b^ (ng/mL)
Agilent 6420	PinPoint Testing, LLC	1–500	0.9991	<1	<1
AbScieX 4500 Q-TRAP	Kentucky State Crime Lab	1–500	0.9997	<1	<1
Agilent 6410	Idaho State Crime Lab	5–250	0.9990	<5	<5
Agilent 6460 (1)	West Virginia State Crime Lab	1–500	0.9954	<1	<1
Agilent 6460 (2)	West Virginia State Crime Lab	1–500	0.9989	<1	<1
AbScieX 4500 Q-TRAP	Ohio State Crime Lab	1–500	0.9980	<1	<1
AbScieX 6600 Triple TOF	Wadsworth Center (NY)	1–500	0.9994	<1	<1
AbScieX 4000 Q-TRAP	Arkansas State Crime Lab	1–100	0.9994	<1	<1

* Data are based on 3 to 6 independent experiments conducted on nonconsecutive days.

a LOD, Lower limit of detection; and defined as the lowest calibrator.

b LOQ, Lower limit of quantification, calculated as 3 times the standard deviation of the lowest calibrator, or as less than the lowest calibrator if estimates are lower

**Table 2:** Summary of linear ranges, correlation coefficients, detection limits, and quantification limits for THC-COOH*

Laboratory/Instrument Comparison for THC-COOH					
Instrument	Laboratory	Linear Working Range (ng/mL)	Average r^2^ value	L.O.D.^a^ (ng/mL)	L.O.Q.^b^ (ng/mL)
Agilent 6420	PinPoint Testing, LLC	5–500	0.9987	<5	<5
AbScieX 4500 Q-TRAP	Kentucky State Crime Lab	5–500	0.9998	<5	<5
Agilent 6410	Idaho State Crime Lab	5–250	0.9951	<5	<5
Agilent 6460 (1)	West Virginia State Crime Lab	5–500	0.9976	<5	<5
Agilent 6460 (2)	West Virginia State Crime Lab	5–500	0.9985	<5	<5
AbScieX 4500 Q-TRAP	Ohio State Crime Lab	5–500	0.9985	<5	<5
AbScieX 6600 Triple TOF	Wadsworth Center (NY)	5–500	0.9992	<5	<5
AbScieX 4000 Q-TRAP	Arkansas State Crime Lab	5–250	0.9991	<5	<5

* Data are based on 3 to 6 independent experiments conducted on nonconsecutive days.

a LOD, Lower limit of detection; and defined as the lowest calibrator.

b LOQ, Lower limit of quantification, calculated as 3 times the standard deviation of the lowest calibrator, or as less than the lowest calibrator if estimates are lower

**Table 3:** Summary of linear ranges, correlation coefficients, detection limits, and quantification limits for THC-OH*.

Laboratory/Instrument Comparison for THC-OH					
Instrument	Laboratory	Linear Working Range (ng/mL)	Average r^2^ value	L.O.D.^a^ (ng/mL)	L.O.Q.^b^ (ng/mL)
Agilent 6420	PinPoint Testing, LLC	5–500	0.9992	<5	<5
AbScieX 4500 Q-TRAP	Kentucky State Crime Lab	5–500	0.9995	<5	<5
Agilent 6410	Idaho State Crime Lab	5–250	0.9955	<5	<5
Agilent 6460 (1)	West Virginia State Crime Lab	1–500	0.9995	<1	<1
Agilent 6460 (2)	West Virginia State Crime Lab	1–500	0.9990	<1	<1
AbScieX 4500 Q-TRAP	Ohio State Crime Lab	1–250	0.9987	<1	<1
AbScieX 6600 Triple TOF	Wadsworth Center (NY)	5–500	0.9996	<5	<5
AbScieX 4000 Q-TRAP	Arkansas State Crime Lab	5–100	0.9998	<5	<5

* Data are based on 3 to 6 independent experiments conducted on nonconsecutive days.

a LOD, Lower limit of detection; and defined as the lowest calibrator.

b LOQ, Lower limit of quantification, calculated as 3 times the standard deviation of the lowest calibrator, or as less than the lowest calibrator if estimates are lower.

**Table 4:** Summary of % Relative Error and One-Sample Mean-Equivalence Test*.

THC					
Concentration	% Relative Error		One-sample mean-equivalence test		
(ng/mL)	Mean (SD)	Min, Max	p-value 1	p-value 2	Conclusion
10	1.9 (11.3)	−10.3, 21.2	0.001	0.0005	Equivalent
25	3.5 (7.0)	−5.6, 15.8	0.0001	<0.0001	Equivalent
100	1.5 (7.9)	−10.0, 14.0	0.0001	0.0001	Equivalent
500^#	0.7 (4.5)	−5.3, 6.7	0.0003	0.0003	Equivalent
THC-COOH					
Concentration	% Relative Error		One-sample mean-equivalence test		
(ng/mL)	Mean (SD)	Min, Max	p-value 1	p-value 2	Conclusion
10	−0.2 (5.5)	−12.6, 5.8	<0.0001	<0.0001	Equivalent
25	−0.2 (3.5)	−5.0, 4.6	<0.0001	<0.0001	Equivalent
100	2.6 (4.7)	−2.8, 10.5	<0.0001	<0.0001	Equivalent
500^	4.0 (2.7)	0.8, 8.0	<0.0001	<0.0001	Equivalent
THC-OH					
Concentration	% Relative Error		One-sample mean-equivalence test		
(ng/mL)	Mean (SD)	Min, Max	p-value 1	p-value 2	Conclusion
10	−0.7 (8.7)	−13.6, 10.3	0.0001	0.0002	Equivalent
25	−0.1 (6.1)	−10.0, 9.4	<0.0001	<0.0001	Equivalent
100	2.8 (6.2)	−6.6, 9.9	0.0001	<0.0001	Equivalent
500^	2.0 (2.6)	−0.5, 5.5	<0.0001	<0.0001	Equivalent

* Four levels of second source quality samples prepared at 10 ng/mL, 25 ng/mL, 100 ng/mL, and 500 ng/mL. One-sample mean-equivalence test was tested against the true concentration value. When both p-values are <0.05, equivalence is determined.

^ 500 ng/mL for all analytes exceeded the working range for Arkansas State Crime Laboratory.

# 500 ng/mL for THC exceeded the working range for Wadsworth Center. Therefore, these data were excluded from statistical analysis.
