# Supplementary material for: Multi-laboratory validation of a Δ9-tetrahydrocannabinol LC-MS/MS test kit designed for quantifying THC and marijuana metabolites in blood
Source: Forensic Sci Criminol. Author manuscript; Available in PMC 2019 Mar 28. (PMC6016857; doi:10.15761/FSC.1000125)
Supplement: Supplementary Tables [file NIHMS962232-supplement-Supplementary_Tables.pdf]

Table S-1. Instrument configuration for Agilent 6410 and 6420.

Parameter	Setting
Mass spectrometer mode	Positive electrospray ionization, dynamic multiple reaction monitoring (dMRM)
Gas Temperature	350 ºC
Gas Flow	12 L/min [Nitrogen]
Nebulizer Gas	50 psi [Nitrogen]
Capillary Voltage	3500 V
Cell Accelerator Voltage	4 V

Table S-2. Instrument configuration for Agilent 6460.

Parameter	Setting
Mass spectrometer mode	Positive electrospray ionization, dynamic multiple reaction monitoring (dMRM)
Gas Temperature	320 ºC
Gas Flow	10 L/min [Nitrogen]
Nebulizer	30 psi [Nitrogen]
Sheath Gas Temperature	320 ºC
Sheath Gas Flow	12 L/min [Nitrogen]
Capillary Voltage	3500 V
Cell Accelerator Voltage	4 V

Table S-3. Instrument configuration for ABSciex 4000 QTrap.

Parameter	Setting
Mass spectrometer mode	Positive electrospray ionization, multiple reaction monitoring (MRM)
Curtain Gas (CUR)	25 cm/S [Nitrogen]
Nebulizer Gas (GS1)	40 cm/S [Nitrogen]
Turbo Gas (GS2)	45 cm/S [Nitrogen]
GS2 Temperature (TEM)	500°C
Interface Heater (ihe)	ON
Collision Gas (CAD)	"high' [Nitrogen]
Ionspray Potential (IS)	1300 V
Entrance Potential (EP)	5 V

Table S-4. Instrument configuration for ABSciex 4500 QTrap.

Parameter	Setting
Mass spectrometer mode	Positive electrospray ionization, multiple reaction monitoring (MRM)
Curtain Gas (CUR)	25 psi [Nitrogen]
Nebulizer Gas (GS1)	65 psi [Nitrogen]
Turbo Gas (GS2)	50 psi [Nitrogen]
GS2 Temperature (TEM)	550ºC
Interface Heater (ihe)	ON
Collision Gas (CAD)	High
Ionspray Potential (IS)	2500 V
Entrance Potential (EP)	5 V

Table S-5. Instrument configuration for ABSciex 6600 Triple TOF.

Parameter	Setting
Mass spectrometer mode	Positive electrospray ionization, DuoSpray Ion Source, in SWATH mode
Curtain Gas (CUR)	25 L/min
Nebulizer Gas (GS1)	45 L/min
Turbo Gas (GS2)	45 L/min
GS2 Temperature (TEM)	350 ºC
Collision Gas (CAD)	25
Ionspray Potential (IS)	4500 V

Table S-6. Mass spectrometry parameters for Specific Reaction Monitoring (SRM) experiments for Agilent 6410, 6420, and 6460.

Analyte	Q1 (m/z)	Q3 (m/z)	Fragmentor (V)	Collision Energy (V)
THC	315.2	193.1*	136	21
		123.1#	136	23
THC-d3	318.2	196.1	132	25
THC-COOH	345.2	327.2*	128	13
		299.1#	128	17
THC-COOH-d9	354.2	336.2	136	13
THC-OH	331.2	313.2*	120	13
		193.1#	120	29
THC-OH-d3	334.3	316.2	115	9

Table S-7. Mass spectrometry parameters for Specific Reaction Monitoring (SRM) experiments for ABSciex 4000 QTrap and 4500 QTrap.

Analyte	Q1 (m/z)	Q3 (m/z)	Declustering Potential (V)	Collision Energy (V)	Cell Exit Potential (V)
THC	315.2	193.1*	35	32	10
		123.1#	35	40	10
THC-D3	318.2	196.1	106	31	8
THC-COOH	345.2	327.2*	106	21	16
		299.1#	106	27	8
THC-COOH-D9	354.2	336.2	106	23	4
THC-OH	331.2	313.2*	71	19	16
		193.0#	71	35	6
THC-OH-D3	334.2	316.1	71	21	14

*: Quantifier ion; #: Qualifier ion

Table S-8. Mass spectrometry parameters for Specific Reaction Monitoring (SRM) Experiments for ABSciex 6600 Triple TOF.

Analyte	Q1 (m/z)	Q3 (m/z)	Collision Energy (V)	Entrance Potential (V)	Declustering Potential (V)	Cell Exit Potential (V)
THC	315.2	193.1*	25	N/A	40	N/A
		123.1#	25	N/A	40	N/A
THC-d3	318.2	196.1	25	N/A	40	N/A
THC-COOH	345.2	327.2*	25	N/A	40	N/A
		299.1#	25	N/A	40	N/A
THC-COOH-d9	354.2	336.2	25	N/A	40	N/A
THC-OH	331.2	313.2*	25	N/A	40	N/A
		193.1#	25	N/A	40	N/A
THC-OH-d3	334.3	316.2	25	N/A	40	N/A

*: Quantifier ion; #: Qualifier ion

Table S-9. Summary of accuracy and precision measurements for PinPoint Testing, LLC.*

	QC High 500ng/mL			*QC Mid2 100/mL*			*QC Mid1 25ng/mL*			*QC Low 10ng/mL*		
	Conc.±SD	%CV[a]	%RE[b]	Conc.±SD	%CV[a]	%RE[b]	Conc.±SD	%CV[a]	%RE[b]	Conc.±SD	%CV[a]	%RE[b]
THC	490.99 ± 44.2	9.0	1.8	98.84 ± 7.9	8.0	2.2	24.36 ± 1.6	6.6	2.6	9.32 ± 0.8	8.6	6.8
THC-OH	497.59 ± 27.0	5.4	0.5	99.61 ± 5.9	5.9	0.4	24.89 ± 1.3	5.1	0.4	9.91 ± 0.8	8.6	0.9
THC-COOH	510.02 ± 18.4	3.6	2.0	97.21 ± 3.4	3.5	2.8	24.69 ± 0.9	3.5	1.2	9.97 ± 0.7	7.2	0.3

*Data obtained using an Agilent 6420.
[a] %CV, Coefficient of Variation, Calculated as ([standard deviation/mean]*100) at each concentration; [b] %RE, Absolute Relative Error, Calculated as (([expected concentration-calculated concentration]/expected concentration)*100) at each concentration

Table S-10. Summary of accuracy and precision measurements for Idaho State Crime Laboratory*..

	QC High 500ng/mL			*QC Mid2 100/mL*			*QC Mid1 25ng/mL*			*QC Low 10ng/mL*		
	Conc.±SD	%CV[a]	%RE[b]	Conc.±SD	%CV[a]	%RE[b]	Conc.±SD	%CV[a]	%RE[b]	Conc.±SD	%CV[a]	%RE[b]
THC	-	-	-	90.03 ± 4.1	4.5	9.9	26.40 ± 0.7	2.7	5.6	11.65 ± 0.5	4.1	16.5
THC-OH	-	-	-	107.98 ± 6.0	5.6	7.9	24.78 ± 1.8	7.1	0.8	11.03 ± 2.3	20.9	10.3
THC-COOH	-	-	-	104.82 ± 4.5	4.3	4.8	23.76 ± 1.8	7.4	4.9	10.58 ± 1.9	18.4	5.8

*Data obtained using an Agilent 6410.
[a] %CV, Coefficient of Variation, Calculated as ([standard deviation/mean]*100) at each concentration; [b] %RE, Absolute Relative Error, Calculated as (([expected concentration-calculated concentration]/expected concentration)*100) at each concentration

Table S-11. Summary of accuracy and precision measurements for Kentucky Crime Laboratory.*

	QC High 500ng/mL			*QC Mid2 100/mL*			*QC Mid1 25ng/mL*			*QC Low 10ng/mL*		
	Conc.±SD	%CV[a]	%RE[b]	Conc.±SD	%CV[a]	%RE[b]	Conc.±SD	%CV[a]	%RE[b]	Conc.±SD	%CV[a]	%RE[b]
THC	510.96 ± 25.7	5.0	2.2	100.48 ± 7.9	4.5	0.5	25.64 ± 1.6	5.1	2.6	10.28 ± 0.5	4.6	2.8
THC-OH	520.20 ± 25.3	4.3	4.0	108.38 ± 8.8	8.1	8.4	26.29 ± 1.9	7.1	5.2	10.48 ± 0.7	6.7	4.8
THC-COOH	517.59 ± 26.6	5.1	3.5	102.78 ± 4.7	4.6	2.8	26.15 ± 1.4	5.5	4.6	10.33 ± 0.6	6.1	3.3

*Data obtained using an ABSciex 4500 QTrap.
[a] %CV, Coefficient of Variation, Calculated as ([standard deviation/mean]*100) at each concentration; [b] %RE, Absolute Relative Error, Calculated as (([expected concentration-calculated concentration]/expected concentration)*100) at each concentration

Table S-12. Summary of accuracy and precision measurements for West Virginia Crime Laboratory – Instrument 1.*

	QC High 500ng/mL			*QC Mid2 100/mL*			*QC Mid1 25ng/mL*			*QC Low 10ng/mL*		
	Conc.±SD	%CV[a]	%RE[b]	Conc.±SD	%CV[a]	%RE[b]	Conc.±SD	%CV[a]	%RE[b]	Conc.±SD	%CV[a]	%RE[b]
THC	508.18 ± 51.1	9.3	1.6	99.27 ± 6.3	6.2	0.7	25.15 ± 1.6	6.3	0.6	9.59 ± 0.5	6.4	4.1
THC-OH	504.87 ± 17.9	3.5	0.9	95.40 ± 3.3	3.5	4.6	23.70 ± 1.4	5.9	5.2	9.50 ± 0.5	5.7	5.0
THC-COOH	515.49 ± 47.6	9.2	3.1	97.80 ± 5.1	5.2	2.2	25.32 ± 1.7	6.8	1.3	9.81 ± 1.4	14.3	1.9

*Data obtained using an Agilent 6460.
[a] %CV, Coefficient of Variation, Calculated as ([standard deviation/mean]*100) at each concentration; [b] %RE, Absolute Relative Error, Calculated as (([expected concentration-calculated concentration]/expected concentration)*100) at each concentration

Table S-13. Summary of accuracy and precision measurements for West Virginia Crime Laboratory – Instrument 2.*

	QC High 500ng/mL			*QC Mid2 100/mL*			*QC Mid1 25ng/mL*			*QC Low 10ng/mL*		
	Conc.±SD	%CV[a]	%RE[b]	Conc.±SD	%CV[a]	%RE[b]	Conc.±SD	%CV[a]	%RE[b]	Conc.±SD	%CV[a]	%RE[b]
THC	533.68 ± 13.2	2.5	6.7	97.30 ± 3.4	3.5	2.7	23.59 ± 1.4	5.9	5.6	8.97 ± 0.8	8.7	10.3
THC-OH	500.81 ± 11.2	2.2	0.2	93.43 ± 2.9	3.1	6.6	22.51 ± 1.1	5.1	9.9	8.64 ± 0.5	6.0	13.6
THC-COOH	539.91 ± 14.8	2.7	7.9	97.67 ± 2.3	2.4	2.3	24.27 ± 1.8	7.4	0.1	10.19 ± 1.2	12.2	1.9

*Data obtained using an Agilent 6460.
[a] %CV, Coefficient of Variation, Calculated as ([standard deviation/mean]*100) at each concentration; [b] %RE, Absolute Relative Error, Calculated as (([expected concentration-calculated concentration]/expected concentration)*100) at each concentration

Table S-14. Summary of accuracy and precision measurements for Ohio Crime Laboratory.*

	QC High 500ng/mL			*QC Mid2 100/mL*			*QC Mid1 25ng/mL*			*QC Low 10ng/mL*		
	Conc.±SD	%CV[a]	%RE[b]	Conc.±SD	%CV[a]	%RE[b]	Conc.±SD	%CV[a]	%RE[b]	Conc.±SD	%CV[a]	%RE[b]
THC	473.59 ± 36.5	7.7	5.3	99.62 ± 7.2	7.2	0.4	25.27 ± 1.4	5.5	1.1	10.04 ± 0.9	8.6	0.4
THC-OH	-	-	-	109.88 ± 6.5	5.9	9.9	27.34 ± 1.4	5.2	9.4	10.29 ± 0.7	7.2	2.9
THC-COOH	503.76 ± 26.7	5.3	0.8	104.15 ± 4.7	4.5	4.2	26.10 ± 1.3	4.9	4.4	10.14 ± 0.6	6.3	1.4

*Data obtained using an ABSciex 4500 QTrap.
[a] %CV, Coefficient of Variation, Calculated as ([standard deviation/mean]*100) at each concentration; [b] %RE, Absolute Relative Error, Calculated as (([expected concentration-calculated concentration]/expected concentration)*100) at each concentration;

Table S-15. Summary of accuracy and precision measurements for Wadsworth Center.*

	QC High 500ng/mL			*QC Mid2 100/mL*			*QC Mid1 25ng/mL*			*QC Low 10ng/mL*		
	Conc.±SD	%CV[a]	%RE[b]	Conc.±SD	%CV[a]	%RE[b]	Conc.±SD	%CV[a]	%RE[b]	Conc.±SD	%CV[a]	%RE[b]
THC	603.68 ± 33.4	5.6	20.7	113.98 ± 5.5	4.9	14.0	27.59 ± 1.8	6.6	10.4	9.57 ± 1.3	13.9	4.3
THC-OH	527.57 ± 8.4	1.6	5.5	105.19 ± 3.6	3.6	5.2	24.45 ± 0.6	2.4	2.2	8.87 ± 0.4	4.7	11.3
THC-COOH	532.25 ± 40.5	7.6	6.5	105.94 ± 6.2	5.8	5.9	24.28 ± 1.5	6.3	2.9	8.74 ± 0.7	7.7	12.6

*Data obtained using an ABSciex 6600 Triple TOF.
[a] %CV, Coefficient of Variation, Calculated as ([standard deviation/mean]*100) at each concentration; [b] %RE, Absolute Relative Error, Calculated as (([expected concentration-calculated concentration]/expected concentration)*100) at each concentration

Table S-16. Summary of accuracy and precision measurements for Arkansas State Crime Laboratory.*

	QC High 500ng/mL			*QC Mid2 100/mL*			*QC Mid1 25ng/mL*			*QC Low 10ng/mL*		
	Conc.±SD	%CV[a]	%RE[b]	Conc.±SD	%CV[a]	%RE[b]	Conc.±SD	%CV[a]	%RE[b]	Conc.±SD	%CV[a]	%RE[b]
THC	-	-	-	112.10 ± 5.0	4.4	12.1	28.95 ± 1.0	3.5	15.8	12.12 ± 0.5	4.1	21.2
THC-OH	-	-	-	102.57 ± 4.0	3.9	2.6	25.85 ± 1.2	4.7	3.4	10.74 ± 0.6	5.9	7.4
THC-COOH	-	-	-	100.53 ± 11.5	11.4	0.5	25.07 ± 2.3	9.0	0.3	10.11 ± 1.1	9.3	1.1

*Data obtained using an ABSciex 4000 QTrap.
[a] %CV, Coefficient of Variation, Calculated as ([standard deviation/mean]*100) at each concentration; [b] %RE, Absolute Relative Error, Calculated as (([expected concentration-calculated concentration]/expected concentration)*100) at each concentration

Copyright: ©2018 Patton AL. This is an open-access article distributed under the terms of the Creative Commons Attribution License, which permits unrestricted use, distribution, and reproduction in any medium, provided the original author and source are credited.
